# Biomechanics of cutting: sharpness, wear sensitivity and the scaling of cutting forces in leaf-cutter ant mandibles

**DOI:** 10.1098/rstb.2022.0547

**Published:** 2023-12-04

**Authors:** Frederik Püffel, O. K. Walthaus, Victor Kang, David Labonte

**Affiliations:** Department of Bioengineering, Imperial College London, London SW7 2AZ, UK

**Keywords:** insects, herbivory, plant fracture

## Abstract

Herbivores large and small need to mechanically process plant tissue. Their ability to do so is determined by two forces: the maximum force they can generate, and the minimum force required to fracture the plant tissue. The ratio of these forces determines the relative mechanical effort; how this ratio varies with animal size is challenging to predict. We measured the forces required to cut thin polymer sheets with mandibles from leaf-cutter ant workers which vary by more than one order of magnitude in body mass. Cutting forces were independent of mandible size, but differed by a factor of two between pristine and worn mandibles. Mandibular wear is thus likely a more important determinant of cutting force than mandible size. We rationalize this finding with a biomechanical analysis, which suggests that pristine mandibles are ideally ‘sharp’—cutting forces are close to a theoretical minimum, which is independent of tool size and shape, and instead solely depends on the geometric and mechanical properties of the cut tissue. The increase of cutting force due to mandibular wear may be particularly problematic for small ants, which generate lower absolute bite forces, and thus require a larger fraction of their maximum bite force to cut the same plant.

This article is part of the theme issue ‘Food processing and nutritional assimilation in animals’.

## 1. Introduction

Plant-feeding occurs at vastly different scales, from large bulk-feeding mammals to tiny cell-ingesting leaf miners [1]. Despite these differences in scale, all herbivores share the same basic task: they need to mechanically process the plant tissue; if they cannot tear, masticate, cut, pierce or drill into the plant, they cannot feed on it. From a simple mechanical perspective, a necessary condition for plant-feeding is then given by two key forces: the maximum force the animal can generate needs to exceed the minimum force required to fracture the plant tissue [1–3]. How do these forces change with animal size?

Based on a simple scaling argument, the maximum available force is expected to increase in proportion to a characteristic area, or with body mass to the power of two-thirds [4]. However, the scaling of the fracture force is difficult to predict, because it depends on plant-mechanical properties [5–9], on the mode of fracture [3,5] and on the geometry of the cutting, chewing or piercing ‘tool’ in question [10–15]. In the absence of robust theoretical frameworks, fracture forces are often determined experimentally instead (e.g. [12,14,16,17]). A key challenge for such experimental approaches is that studies across a large range of tool sizes typically require using different species, so that fracture tools usually differ in both scale and shape [2,12]. In order to investigate the influence of tool size alone, we here measured fracture forces using the cutting tools of a species for which adults vary substantially in size but only little in shape: *Atta vollenweideri* leaf-cutter ants.

Leaf-cutter ant colonies consist of up to several million workers, which cover a large range of body sizes, from less than a milligram to over 100 mg in some *Atta* species [18–21]. Notably, this size range reflects ‘static’ differences among workers at equivalent developmental stages; workers retain their adult form after eclosion from the pupa. Fully matured leaf-cutter ant foragers cut leaf fragments from plants in the colony surroundings; these fragments are then carried back to the colony to grow a subterranean fungus as crop [22–25]. To cut transportable fragments from large leaves, leaf-cutter ants typically use one of their mandibles as an ‘anchor’ that pierces through the leaf lamina but remains approximately stationary; a single cut is then made by drawing the second mandible through the leaf lamina like a blade ([26,27], see electronic supplementary material, video). Repeated cutting cycles, combined with a ‘pivoting’ of the ant around an approximately fixed anchor point for the hind legs, then yields leaf fragments with semi-circular shape that can be carried back to the nest (e.g. [26,28]). Interestingly, the tendency to cut and carry plant fragments correlates with worker size: larger ants cut and carry larger fragments [19,28–30], at higher speeds [27,30–35] and forage on ‘tougher’ plants than smaller ants [23,24,28,31,33,36–38].

By contrast to this robust empirical evidence for size-related preferences in foraging, the biomechanical factors that underpin it remain poorly understood (but see [27,28,39]). For example, do larger workers cut tougher leaves because smaller workers are unable to do so, or because they cut more efficiently? In order to assess how the ability to cut leaves varies with size, we previously measured maximum bite forces of *A. vollenweideri* leaf-cutter ants [40]. Peak bite forces increased with strong positive allometry, *F*_b_ ∝ *m*^0.90^, in substantial excess of the isometric prediction, *F*_b_ ∝ *m*^0.67^: a large forager of 40 mg generates peak bite forces of about 800 mN, 16 times more than a small forager of 2 mg, *F*_b_ ≈ 50 mN, and about as large as the bite forces of a vertebrate 20 times heavier [40]. As a result, large foragers are presumably able to cut a considerably larger fraction of tropical leaves [8,40].

However, this conclusion is premature, because it remains unclear how the forces required to cut vary with mandible size. For example, one may speculate that cutting forces vary with a characteristic length (e.g. [12,41,42]); smaller ants would then have ‘sharper’ mandibles, which demand less force to cut a given material. To complicate matters further, mandible ‘sharpness’ may vary across the lifetime of an ant owing to mandibular wear [27]. The degree of mandibular wear likely depends on the abrasiveness of the cut materials [43,44], the wear resistance of the mandible teeth [45–47], the mandible tooth geometry [2,12] and the forces involved in cutting [12]. To investigate the impact of mandibular wear on cutting forces and to compare it with the impact of mandible size, we performed cutting force experiments using mandibles either from freshly eclosed ants (callows), which initially remain in the nest and thus have ‘pristine’ mandibles, or from workers that actively partook in foraging, and thus are likely to have worn mandibles. We hypothesize (i) that pristine mandibles of small ants cut with less force than pristine mandibles of large ants because they are sharper, and (ii) that forager mandibles cut with larger forces compared with callow mandibles of the same size, as they are blunted by wear.

## 2. Material and methods

### (a) Study animals

We sampled *A. vollenweideri* leaf-cutter ants from two colonies, founded and collected in Uruguay in 2014. The colonies were kept in a climate chamber (FitoClima 12.000 PH, Aralab, Rio de Mouro, Portugal) at $25^{\circ }C$ and 50–60% relative humidity, with a 12/12 h light–dark cycle. They were provided with bramble, laurel, maize and honey water ad libitum, supplied in a foraging arena that was connected to the main colony via PVC tubes (*ca* 30 cm to the closest fungus box; 25 mm inner tube diameter).

To quantify how the force required to cut thin leaf-like sheets varies with mandible size, we collected two sets of ants across the worker size-range, excluding the smallest workers, which typically do not cut leaves (body mass less than 1 mg, see [24,48]). First, we extracted workers from the fungal garden that either had eclosed recently, as indicated by their bright cuticle, or were still in the pupal stage (*n* = 46, [27,49]). In the weeks following eclosion, callows remain inside the nest and abstain from foraging activities [24,50]. The mandibles of callow workers are thus likely ‘pristine’, which allowed us to test for the effect of mandible size on cutting force without potentially confounding effects due to mandibular wear [27]. To ensure that the incorporation of reinforcing zinc into the mandibular teeth was completed, callows were kept alive for at least 72 h post eclosion, defined as time point at which the legs had completely unfolded [27,49]. To monitor pupae and callows, they were placed in centrifuge tubes, which in turn were kept inside the foraging arena. The tubes contained small amounts of fungus, and had a 3D-printed polylactic acid (PLA) lid with holes too small for the collected workers to pass through, but large enough for minims to enter for pupal maintenance [27]. This method was thus unsuitable for smaller ants (less than 10 mg), which were collected by transferring late-stage pupae into a separate container with sufficient amounts of fungus and numerous minims instead. Hatched ants were marked with a unique colour code (Edding 4000 paint marker, Edding, Ahrensburg, Germany; [51]).

Second, we collected fully matured workers from the foraging arena (*n* = 39). These workers were likely to have mandibles worn from the repeated cutting of leaves [27]. Quantifying the mandibular cutting forces for these active workers allowed us to investigate the effect of mandibular wear and its interaction with worker size.

### (b) Mandible preparation and wear quantification

**Figure 1.**
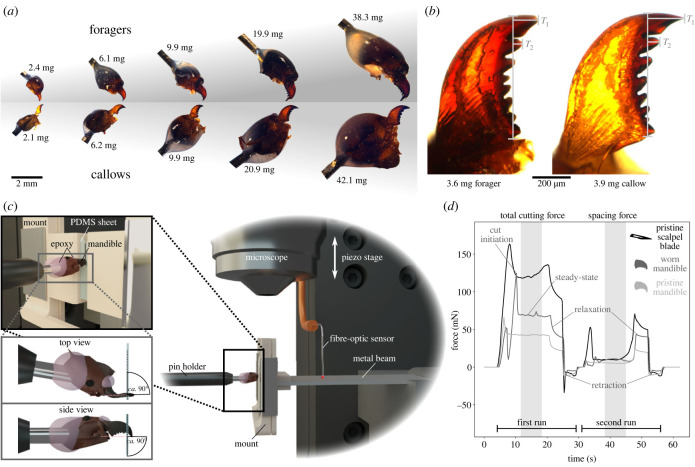
(*a*) In order to measure mandibular cutting forces, *Atta vollenweideri* leaf-cutter ants were extracted from the foraging arena, or from the fungal garden of mature colonies (body mass: 1.8–46.4 mg). Both foragers (with worn mandibles) and callows (with pristine mandibles) were collected to quantify the effects of worker size and mandibular wear on cutting force in isolation. (*b*) For each mandible, we calculated a wear index based on absolute length changes relative to the most and second most distal teeth, *T*_1_ and *T*_2_, respectively (see [27], and electronic supplementary material for more details). (*c*) Cutting forces were then measured using a custom-built setup based on a fibre-optic displacement sensor and a bending beam, both connected to a piezo motor stage. A polydimethylsiloxane (PDMS) sheet was fixed in a custom-designed holder, mounted at the free end of the beam, and the mandible was positioned above the sheet such that its cutting edge was perpendicular to the sheet plane. The motor then moved the beam mounting vertically against the mandible, causing the sheet to be cut and the beam to deflect. (*d*) After an initial loading phase, cutting force peaked at cut initiation and then dropped to an approximately constant value. At the end of this ‘steady-state’ phase, the forces dropped again, when the motor stopped, and became negative as the setup was moved back to its original position. A second run through the cut was performed in the same position to extract the spacing force arising from sheet bending and friction (e.g. [11,42]). The drift-corrected average total cutting force and the corresponding spacing force across 2 mm cutting distance (shaded areas) were extracted for statistical analysis.

All ants were sacrificed by freezing, weighed to the nearest 0.1 mg (Explorer Analytical EX124, max. 120 g × 0.1 mg, OHAUS Corporation, Parsippany, NJ, USA; body masses between 1.8 and 46.4 mg), and decapitated using micro-scissors. The head capsules were split in half along the sagittal plane using a scalpel, and only the left head hemisphere was retained (figure 1*a*). Leaf-cutter ants show no preference between left and right mandible when cutting [26], and their bite apparatus is bilaterally symmetric [52]. We hence assume that there are no systematic differences between left and right mandibles. To facilitate sample mounting, insect pins were inserted into the head halves (size ‘2’ for ants less than 10 mg, size ‘4’ for ants of 10–20 mg and size ‘6’ for ants greater than 20 mg; Shigakontyu, Tokyo, Japan). The interface between insect pin, head capsule and mandible base was then immobilized with two-component epoxy to minimize compliance of the mandible–head–pin complex (Araldite Rapid, Huntsman Corporation, The Woodlands, TX, USA; see figure 1*a*).

In order to determine a proxy for the degree of mandibular wear, all mandibles were photographed with a camera mounted onto a light microscope, such that their dorsal surface was in focus (DMC5400 on Z6 Apo, Leica Microsystems, Wetzlar, Germany; see figure 1*b*). Numerous empirical metrics for mandibular wear have been proposed in the literature, including variation of mandible length [53,54], shape changes of the mandibular cutting edge [55–57], number of lost mandibular teeth [58], reduction in profile area of distal mandibular teeth [59] and length changes of the mandibular teeth most relevant for cutting [27,60]. All of these metrics are proxies, with no direct established mechanistic relation to cutting force. As such, their predictive value can only be assessed in correlation to direct cutting force measurements, and selecting any one of them is difficult to justify *a priori*. We chose a metric that has been demonstrated to correlate significantly with cutting force in closely related *Atta cephalotes* ants, so enabling a direct comparison [27]; however, we do not intend to imply that this metric is more or less predictive than any of the others. Following Schofield *et al.* [27], a mandibular wear index, *W*, may be defined as:

2.1 $$W=\frac{\left(\Delta T_{2}+\alpha \Delta T_{1}\right)}{2}.$$

Here, Δ*T*_1_ and Δ*T*_2_ are the differences between observed and pristine tooth length for the most distal and second most distal tooth, respectively; *α* is a weighting factor, defined as ratio between the average length differences, $\alpha =\bar{\Delta T_{2}}/\bar{\Delta T_{1}}$ (for more details, see [27]). This wear index has dimension length, and may be interpreted as the weighted average length change of the two distal-most teeth; it is thus a proxy for absolute rather than relative wear.

To calculate the wear index, the lengths of the mandible blade and the two most distal teeth were measured from each photograph (figure 1*b*), and the pristine tooth length expected for the same body mass was estimated from a regression analysis on measurements of callow mandibles (for exact methodology, see electronic supplementary material). The wear index could not be extracted for 17 out of 76 mandibles, because relevant parts of the mandible were obstructed by the head capsule.

### (c) Cutting force setup

Mandibular cutting forces were measured with a custom-built setup based on a fibre-optic displacement sensor (μDMS−RC32 controlled via DMS Control v. 3.015, Philtec, Annapolis, MD, USA; linear range of 2.5 mm, recording at 81.4 Hz at $30^{\circ }$C and 50% transmitted optical power). The sensor was held in place by a custom-built holder, mounted on two micromanipulators to control its orientation, and attached to a piezo motor stage (M-404.6PD controlled via PIMikroMove v. 2.33.3.0, Physik Instrumente, Karlsruhe, Germany; see figure 1*c*). The sensor was placed above a stainless steal bending beam with a thickness of 0.40 mm, a width of 10.4 mm and a free length of 28.7 mm, such that the sensor tip was about 400 μm above the beam surface (see [61], for a similar setup). The beam was clamped to the motor stage at one side. At the free end of the beam, a 3D printed mount was attached. This mount held the cutting substrate during the experiments, clamped in place by two metal clips (Supaclip 40, Rapesco Office Products, Sevenoaks, UK). At the centre of the mount, there was a ‘free’ cutting region, 1.5 mm wide and 8 mm long, over which mandibles or scalpel blades were positioned for cutting experiments (figure 1*c*).

The sensor was calibrated with ten calibration weights ranging between 10 and 245 mN (1–25 g, Kern & Sohn, Balingen, Germany), covering the range of observed total cutting forces (19–172 mN). Weights were suspended from the mount in increasing order, and at the lever arm at which cutting forces were applied. For each calibration weight, we averaged the sensor output across 5 s after initial force fluctuations had faded (see electronic supplementary material, figure S2C). To account for sensor drift, the sensor output was extracted for the unloaded beam at the beginning and end of the measurement, and a linear drift correction was implemented; sensor drift, however, was generally small, *ca* 0.01 mN s^−1^, or 6 mN min^−1^, and thus less than 5% of the smallest total cutting force over the duration of a typical measurement of 60 s. From simple beam theory, the relationship between applied force and beam deflection should be linear for small deflections. We indeed observed linearity for calibration forces less than 150 mN; for forces exceeding 150 mN, however, the sensor distance was systematically ‘sub-linear’, suggesting deflections sufficiently large to invalidate the use of the small angle approximation. We thus used a quadratic regression model to characterize the relationship between force and distance, which accounted for more than 99% of the variation, and yielded a lower Akaike information criterion compared with either a linear or a cubic model (AIC_linear_ = 91.7, AIC_quadratic_ = 59.6, AIC_cubic_ = 61.6; see electronic supplementary material, figure S2D).

### (d) Polymer sheet production and mechanical testing

Previous studies on mandibular cutting forces used leaf lamina and floral petals as cutting substrates [26,27]. This choice has the advantage that it is of direct biological relevance. However, plant tissues are typically heterogeneous, of uneven thickness, and have mechanical properties that vary with hydration and tissue age, so introducing covariation that is difficult to control (e.g. [9,62–65]). In order to minimize variation due to material inhomogeneities, we used well-defined polydimethylsiloxane (PDMS) sheets as cutting substrate.

PDMS sheets were made with a 4 : 1 (silicon base : curing agent) mixing ratio (SYLGARD 184, Dow, Midland, MI, USA). The mixed but uncured PDMS was sandwiched between two silanized glass plates, separated by feeler gauges (200 μm, Precision Brand, Downers Grove, IL, USA; see electronic supplementary material, figure S2A), and pre-cured in an oven at $100^{\circ }C$ for 2 h (Drying oven, Sanyo Electric Co., Osaka, Japan). The PDMS ‘sandwich’ was then cooled to room temperature, slowly peeled from the glass plates, placed on aluminium foil and fully cured at $165^{\circ }C$ for a further 48 h [66]. Sheet thickness was verified through measurement at six random locations across the sheet with a digital micrometer (max. 25 × 0.001 mm, Mitutoyo Corporation, Kawasaki, Japan), and was 215 ± 8 μm (mean ± standard deviation), or within 10% of the target thickness.

To mechanically characterize the PDMS sheets, pure shear tearing and uniaxial tension tests were conducted with a universal tension and compression system (Multitest5-xt, Mecmesin, Slinfold, UK; 10 N load cell and Mec277 double-action vice grips with diamond jaws). Two rectangular samples from each of the eight PDMS sheets were cut and used for pure shear tearing; in one of the two paired samples, a notch of 3 mm length was introduced at the centre of the short side (see electronic supplementary material, figure S2A for dimensions). Both samples were tested at a low strain rate of 0.0067 s^−1^ (motor speed divided by sample height) to approximate quasi-static loading conditions. The critical displacement to rupture was then extracted from the notched sample based on a time-synchronized video recording. The force–distance curve of the unnotched sample was integrated from zero to this critical displacement to obtain the work done by the applied load. Fracture toughness was then calculated as this work divided by sample width and thickness (for more details, see [67,68]), yielding an average of *G*_c_ = 98 ± 7 J m^−2^ (for comparison, see [68]).

Next, uniaxial tension tests at 0.5 mm s^−1^ motor speed were conducted with two ‘dog-bone’ samples cut from each of eight sheets according to ISO37 and ISO5893. The Young’s modulus was extracted from the loading region of the stress–strain curve via linear regression between 0 and 10% strain [69,70]; on average, the Young’s modulus was *E* = 4.1 ± 0.3 MPa, in good agreement with published estimates [66].

### (e) Cutting experiments

Individual ant heads were fixed onto a pin holder, which was connected to a 3D micromanipulator (*n* = 85; Manipulator MM 33, Märzhäuser Wetzlar, Wetzlar, Germany). The mandibles were then positioned using a top-down microscope, such that the dorsoventral head axis was approximately horizontal, the mandibular teeth were roughly perpendicular to the PDMS sheet, and the most distal tooth tip just about extended over the sheet edge (see [27] and figure 1*c*).

We cut a small wedge into all PDMS sheets ($ca.\;30^{\circ }$ and 1.5 mm deep) to facilitate cut initiation by reducing effects of sheet bending and buckling [27,68,71]. The polymer sheets were then placed individually between the two components of the polymer mount, and metal clips were slid onto the mount using the clip dispenser provided by the manufacturer, such that both clamps were approximately parallel and 6 mm away from the mount centre (figure 1*c*); this procedure ensured that the clamping conditions were kept approximately constant across measurements.

The beam mount was then moved toward the mandible until the tip of the pre-cut wedge was about to contact the mandibular cutting edge. The sensor recording was started, and the beam mount was moved vertically against the mandible blade, resulting in cutting motion somewhat akin to the ‘blade-like’ cutting behaviour observed in freely cutting leaf-cutter ants [26,27]. The motor moved at a constant speed of 0.3 mm s^−1^, at the upper end of cutting speeds observed during foraging (*ca* 0.02−0.30 mm s^−1^ [23,26,27,30,33,34,72]), and over a total distance of 5 mm; the beam deflected by around 100 μm for a medium cutting force of 65 mN, so that the corresponding displacement of the sheet-holding mount was about 4.9 mm (see electronic supplementary material, figure S2C,D). The sheet was subsequently retracted to its original position, and a second run was initiated in order to extract the force due to elastic sheet deformation and sidewall friction (henceforth referred to as spacing force, e.g. [11,42,73,74]). After a force peak at cut initiation, the total cutting force dropped and remained approximately constant until the motor stopped (figure 1*d*). We extracted the drift-corrected steady-state total cutting force averaged across 2 mm following the initial peak; the corresponding spacing forces were extracted from the second run at the same motor positions, and averaged across the same distances (figure 1*d*).

Cutting speeds typically vary with forager size; larger ants cut more quickly [27,33,34]. The effects of speed on cutting force depend on the viscoelastic properties of the material, but are typically small for elastomers such as PDMS cut at low rates [68,75]. To briefly confirm that the speed-dependency is indeed small, we performed a series of measurements at 0.1, 0.2 and 0.3 mm s^−1^ motor speed, using the mandible of a single forager with a body mass of 19.9 mg. Three repetitions were completed per speed, without remounting the mandible between measurements, to reduce confounding effects due to small variations in mandible blade orientation. This variation was also quantified, by measuring cutting forces of one small (5.4 mg) and one large forager mandible (38.4 mg) at a constant cutting speed of 0.3 mm s^−1^. Both samples were mounted three times onto the pin holder, and cutting experiments were performed three times per mount.

Mounting had no significant effect on total cutting force (analysis of variance (ANOVA), small worker: *F*_2,6_ = 4.43, *p* = 0.07; ANOVA, large worker: *F*_2,6_ = 0.26, *p* = 0.71); we hence pooled the nine measurements per mandible and calculated the coefficients of variation, CV_small_ = 0.10 and CV_large_ = 0.03 (see electronic supplementary material, figure S1). The relative force variation was significantly larger for the smaller mandible (asymptotic test for equality of CV: *D*_AD_ = 7.76, *p* < 0.01, implemented in the R package ‘cvequality’, v. 0.2.0, [76]), suggesting that consistent mandible alignment is easier for larger mandibles. However, even for the smaller mandible, the force variation was small in comparison with the inter-individual variation across all foragers, CV_foragers_ = 0.52 (see below). We thus performed only a single measurement per specimen, unless otherwise indicated.

To contextualize our results based on *biological* ant mandibles and *synthetic* PDMS sheets, we performed two additional experiments. First, we measured cutting forces of pristine scalpel blades (carbon steel, no. 11, Swann-Morton, Sheffield, UK), positioned such that the blade tip just about extended over the sheet edge to reduce the contact area with the PDMS sheet (*n* = 5). Second, we performed cutting experiments with mandibles on a biological substrate, the leaf lamina of Japanese laurel, *Aucuba japonica*; the colonies were regularly fed with these leaves, and the lamina appeared comparatively homogeneous. Laurel leaves were cut from the plant on the day of the experiment, and kept hydrated using wet tissues between collection and measurement. To reduce variation due to material inhomogeneities, we cut all laurel samples from the same plant, from a leaf region close to the mid-vein. Prior to the cutting experiment, we measured lamina thickness and mounted the samples such that the cut ran perpendicular to the mid-vein. We used mandibles of 13 out of the 85 prepared ants, seven foragers (body mass 5.4–38.8 mg) and six callows (body mass 6.2–46.4 mg), mounted once with one to three repetitions per specimen. To account for differences in lamina thickness, *t*_l_, we corrected the measured total cutting force, *F*_m,c_, as $F_{c}=F_{m,c}\bar{t_{l}}/t_{l}$, where $\bar{t_{l}}$ was the average lamina thickness (256 ± 29 μm).

Across all experiments, measurements were considered invalid and thus repeated when at least one of the following criteria was met: (i) the head capsule came into contact with the clamp or the cutting substrates; this occurred when the head capsule was initially close to the clamp and the PDMS sheet buckled; (ii) the mandible slipped out of the cut; (iii) the steady-state phase was too short to extract a meaningful cutting force (less than 2 mm); (iv) the epoxy fixation of the joint failed, leading to mandible rotation (in these cases, the samples were re-glued and used again); and (v) the sample slipped out of the pin holder, as observed occasionally for measurements involving high cutting force (see below).

### (f) Data curation and statistical analysis

We excluded a total of four out of 46 callows and five out of 39 foragers, because optical inspection of the mandible suggested that small amounts of epoxy contaminated the mandibular teeth, and cleaning attempts failed or caused visible damage. Additionally, we excluded one out of seven forager–laurel measurements, because the total cutting force exceeded the calibration range (greater than 245 mN).

Extraction of the average total cutting and spacing force from the raw data was done in Python (v. 3.9.7 [77]), and all statistical analyses were conducted in R (v. 4.1.1 [78]). To characterize the relationship between the extracted forces, body mass, and the two experimental groups (foragers versus callows), we used analysis of covariance (ANCOVA) with Type III sums of squares [79]. In addition, we performed ordinary least squares (OLS) regressions to characterize the scaling relationships within the experimental groups. Unless stated otherwise, we performed these analyses on log_10_-transformed data.

## 3. Results

### (a) Cutting forces are independent of mandible size

**Figure 2.**
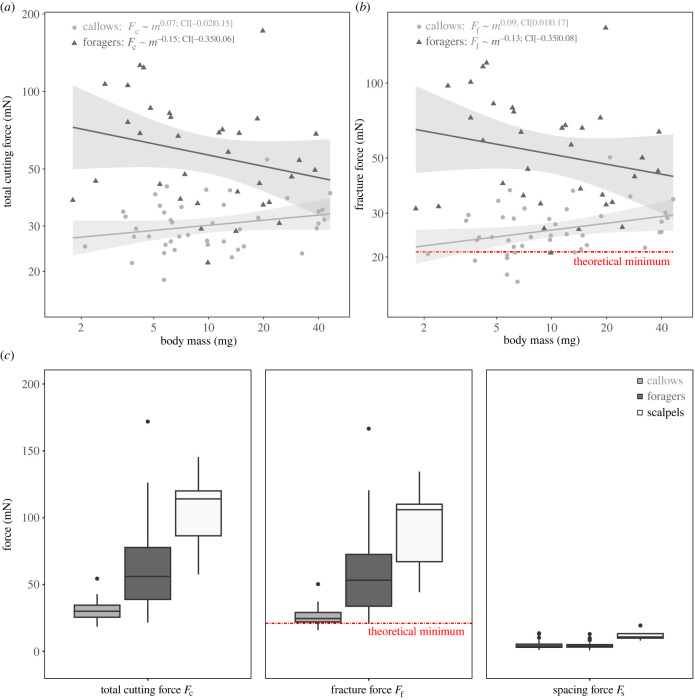
(*a*) Leaf-cutting is performed by workers spanning approximately one order of magnitude in body mass. To assess how cutting ability is affected by body mass, we measured mandibular cutting forces across almost the entire size range (body mass, *m*, 1.8–46.4 mg), and across two experimental groups: callows with pristine mandibles (*n* = 42), and active foragers with mandibles affected to varying degrees by wear (*n* = 34). Total cutting forces, *F*_c_, were independent of body mass for both experimental groups (see main text for statistics), but about twice as high for foragers compared with callows. (*b*) Fracture forces, *F*_f_, were not significantly affected by body mass in foragers. For callows, however, *F*_f_ increased significantly, *F*_f_ ∝ *m*^0.09^, from values close to a theoretical minimum predicted via biomechanical modelling to values closer to those obtained from foragers. (*c*) On average, total cutting and fracture forces of both pristine and worn mandibles were significantly smaller than those measured for pristine scalpel blades (*n* = 5, *F*_c_ = 105 ± 34 mN, *F*_f_ = 92 ± 36 mN). Spacing forces, *F*_s_, were about 5 ± 3 mN for both groups independent of body mass, and significantly smaller than for scalpel blades; they contributed only around 10% of the total cutting force for mandibles. All boxplots display the median (centre line), the first and third quartiles (hinges), extended by 1.5 times the interquartile range (whiskers), and outliers (points). (Online version in colour.)

Total cutting forces, *F*_c_, were independent of body mass (ANCOVA: *F*_1,72_ = 0.97, *p* = 0.33), but depended significantly on the experimental group (callows versus forager, *F*_1,72_ = 21.2, *p* < 0.001; see figure 2*a*). These main effects must be interpreted with caution, as the interaction term was significant (*F*_1,72_ = 4.42, *p* < 0.05, see [79]), suggesting that the relationship between total cutting force and body mass differs between the experimental groups. Indeed, within callows, total cutting forces tended to increase with body mass, whereas they decreased slightly within foragers. However, neither result was significant (*p* ≥ 0.11, see table 1).

**Table 1.** Results of ordinary least squares regressions describing the relationship of total cutting force, *F*_c_, spacing force, *F*_s_, fracture force, *F*_f_, absolute and relative mandibular wear index, *W* and $W^{⋆}$, respectively, with body mass in milligrams. All regressions were performed on log_10_-transformed values, apart from mandibular wear, which contained negative values; this regression was done on semi-*log*_10_-transformed data instead. 95% confidence intervals are provided in parentheses. The low *R*^2^ values underline that body size only had a small influence on all performance metrics.

quantity	group	elevation	slope	*R*^2^
*F*_c_ (mN)	foragers	1.90 (1.69, 2.11)	−0.15 (−0.35, 0.06)	0.06
*F*_c_ (mN)	callows	1.41 (1.33, 1.50)	0.07 (−0.02, 0.15)	0.06
*F*_s_ (mN)	foragers	0.74 (0.51, 0.98)	−0.14 (−0.37, 0.08)	0.05
*F*_s_ (mN)	callows	0.65 (0.42, 0.88)	−0.05 (−0.27, 0.16)	0.01
*F*_f_ (mN)	foragers	1.85 (1.62, 2.07)	−0.13 (−0.35, 0.08)	0.05
*F*_f_ (mN)	callows	1.32 (1.23, 1.41)	0.09 (0.01, 0.17)	0.11
*W* (μm)	foragers	13.43 (1.75, 25.10)	−6.02 (−17.19, 5.16)	0.05
$W^{⋆}$	foragers	0.24 (0.07, 0.42)	−0.13 (−0.29, 0.04)	0.10

On average, total cutting forces of forager mandibles exceeded those of callow mandibles by a factor of two, 64 ± 33 versus 31 ± 7 mN, respectively (figure 2*c*). Notably, the coefficient of variation also differed significantly by about a factor of two (CV_foragers_ = 0.52 and CV_callows_ = 0.23; asymptotic test for equality of CV: *D*_AD_ = 19.1, *p* < 0.001), suggesting that both relative and absolute force variation was larger among foragers. The magnitude of total cutting force extracted for both groups was small in comparison with the total cutting force measured with pristine scalpel blades, 105 ± 34 mN, (see figure 2*c*; Wilcoxon rank sum test, forager mandibles versus scalpel blades: *W* = 29, *p* < 0.05; callow mandibles versus scalpel blades: *W* = 0, *p* < 0.001).

Across all ant mandibles, spacing forces, *F*_s_, were 5 ± 3 mN, with neither significant differences between experimental groups, nor significant size-effects (ANCOVA, experimental group: *F*_1,72_ = 0.34, *p* = 0.56; body mass: *F*_1,72_ = 0.25, *p* = 0.62; see table 1 and electronic supplementary material, figure S3D). Because callow mandibles cut with about half the force, the relative spacing component was about two times higher (15±8% versus 8±6% (ANCOVA: *F*_1,72_ = 5.98, *p* < 0.05)). Spacing forces of scalpel blades were 12 ± 4 mN, or 14±8% of the cutting forces, significantly larger than for callow and forager mandibles (see figure 2*c*; Wilcoxon rank sum test, forager mandibles versus scalpel blades: *W* = 8, *p* < 0.001; callow mandibles versus scalpel blades: *W* = 10, *p* < 0.001).

Because mandible spacing forces were size-invariant, the scaling of fracture forces, *F*_f_ = *F*_c_ − *F*_s_, essentially mirrored the results obtained for the total cutting force (figure 2*b*). Fracture forces were independent of body mass (ANCOVA: *F*_1,72_ = 1.64, *p* = 0.20), but depended significantly on experimental group (*F*_1,72_ = 22.6, *p* < 0.001), with a significant interaction (*F*_1,72_ = 4.46, *p* < 0.05). Within foragers, *F*_f_ tended to decrease with size, but this trend was not significant (*p* = 0.22, see table 1); on average, *F*_f_ was 59 ± 32 mN, comparable to the minimum force obtained for scalpel blades (44 mN). Within callows, however, *F*_f_ now increased significantly with worker size (*p* < 0.05, see table 1), at the lower end approaching the minimum cutting forces predicted from tearing experiments (21 mN, see figure 2*b* and Discussion).

**Figure 3.**
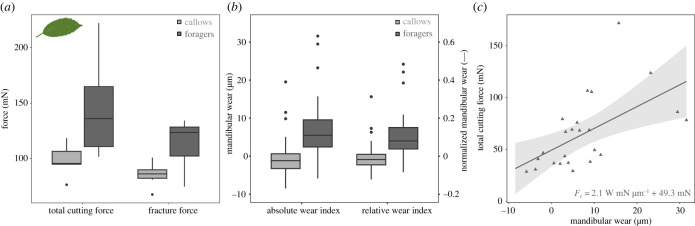
(*a*) We tested if the difference in cutting force between callows and foragers persisted on biological substrates by performing cutting experiments on *Aucuba japonica* leaf lamina, using mandibles from six foragers and six callows across the size range. The absolute difference in fracture force between callows and foragers was 28 mN, similar to the results obtained for polydimethylsiloxane (PDMS) sheets (33 mN). (*b*) We calculated a simple mandibular wear index, defined as the weighted average length change of the two most distal teeth (*n* = 66, see [27] and equation (2.1)). In foragers, the absolute wear index, *W*, was 8 ± 10 μm, independent of worker size; by definition, *W* was centred around zero for callows (0 ± 5 μm). On average, foragers lost approximately 12% of their distal tooth length, as indicated by the relative wear index, $W^{⋆}$. All boxplots display the median (centre line), the first and third quartiles (hinges), extended by 1.5 times the interquartile range (whiskers) and outliers (points). (*c*) In foragers, mandibular cutting forces increased significantly with absolute wear index (*n* = 24, ordinary least squares regression on forager data: slope=2.09,95% CI (0.76|3.43), *R*^2^ = 0.33). Although the total variation explained remains below 50%, the wear index thus accounts for six times more variation than body mass. (Online version in colour.)

To test if the observed differences in mandibular cutting forces between callow and forager mandibles are also present with biological substrates, we measured cutting forces for a small subset from both experimental groups on laurel leaf lamina. Total cutting forces, corrected for differences in lamina thickness, were 141 ± 44 mN for foragers, exceeding those of callows (95 ± 14 mN) by almost 50 mN (figure 3*a*); this difference was not significant (Welch two-sample *t*-test: *t*_5.99_ = −2.40, *p* = 0.054). After subtracting spacing forces, however, the difference in fracture force was significant (114 ± 23 mN versus 85 ± 11 mN; two-sample *t*-test: *t*_10_ = −2.68, *p* < 0.05), and averaged 28 mN, similar to the result obtained for PDMS (33 mN).

### (b) Cutting speed only has a small effect on cutting force

The cutting speeds during natural foraging typically vary with both worker size and leaf mechanical properties; larger ants cut faster than smaller ants, and ‘tougher’ leaves are cut more slowly than ‘tender’ leaves [27,33,34]. We quantified the interaction between speed and total cutting force on synthetic PDMS sheets: total cutting forces increased significantly but modestly with speed (ANOVA: *F*_1,7_ = 33.0, *p* < 0.001) from 29 ± 2 mN at 0.1 mm s^−1^ to 36 ± 1 mN at 0.3 mm s^−1^ (see electronic supplementary material, figure S1). Total cutting forces thus increased by only 20% for a threefold increase in speed.

### (c) Cutting forces increase significantly with mandibular wear

The mean mandibular wear index of foragers was 8 ± 10 μm, significantly different from zero, which defines the pristine state (one-sided Wilcoxon rank sum exact test: *V* = 272, *p* < 0.001), and independent of body mass (ANOVA on semi-log_10_-transformed data: *F*_1,22_ = 1.25, *p* = 0.28; see table 1 and figure 3*b*). This size-independence suggests that absolute wear was the same across sizes, and thus that smaller ants lost a larger fraction of their teeth to wear. Although the relative mandibular wear index, normalized with the pristine length of the second most distal tooth, indeed slightly decreased with size from about 20% for a 3 mg forager to 5% for a 30 mg forager, this decrease was not significant (ANOVA on semi-log_10_-transformed data: *F*_1,22_ = 2.43, *p* = 0.13; see table 1). Total cutting force increased significantly with absolute wear at a rate of 2.09 mN μm^−1^ (OLS regression on untransformed data: 95% CI of slope (0.76|3.43), *p* < 0.01, *R*^2^ = 0.33; see figure 3*c*), comparable to the rate of 3.7 mN μm^−1^ reported for closely related *A. cephalotes* [27].

## 4. Discussion

Leaf-cutter ants are iconic herbivores, with key impact on ecosystem ecology throughout the Neotropics [25,80]. The continuous size variation of their workers has also made them a model system for the study of ergonomic benefits of advanced polyethism in social insects (e.g. [24,36,81–83]). A key task faced by any leaf-cutter colony is to cut plant tissue fragments in the colony surroundings, used in the nest to maintain a fungus used as crop. Workers of which size are best suited for this task? Larger workers generate larger bite forces, and may thus be able to cut a larger variety of leaves [40,52]. But the ability to cut depends not only on the available bite force, but also on the force required to cut the leaf—the key determinant of cutting ability is the ratio between both forces. Larger mandibles are putatively blunter, and may thus require larger bite forces to cut the same material [12–14]. How do cutting forces vary with mandible size?

In this work, we approached this question empirically, and measured the forces required to cut homogeneous PDMS sheets with mandibles of workers across the body size range. Cutting forces varied only weakly with mandible size, but differed considerably between mandibles taken from callows, which were pristine, and mandibles taken from foragers, which were affected to varying degree by wear. Before we discuss the biological implications and mechanical basis of these results, we briefly address two key aspects in which our experiments differ from natural cutting behaviour.

First, one may raise reasonable doubts about the extent to which results obtained on a synthetic elastomer can enable conclusions about biologically relevant cutting performance on leaves. The choice of PDMS as a test substrate was motivated by the need to minimize confounding variation in cutting forces due to material inhomogeneities and age- and hydration-dependence, expected for heterogeneous biological materials such as leaves (e.g. [8,9,62–65]). However, whether mandibles cut PDMS or leaves, the involved forces are amenable to mechanical analysis from first principles. We provide such an analysis at the end of the discussion, and the results confirm that the main conclusions of our study likely carry over to biological substrates, so enabling an initial discussion that focuses on biological implications.

Second, we acknowledge that even if experiments with PDMS can provide insights into cutting forces expected for leaves, our cutting experiments do not fully mirror the complexity of cutting behaviour of leaf-cutter ants. For example: mandibles rotate instead of translate; neck muscles may be used to change head and mandible orientation during cutting, and perhaps even directly contribute to cut propagation; and the section of the mandible blade used for cutting may be adjusted to account for local differences in mandible ‘sharpness’, or to dynamically alter the effective mechanical advantage of the mandible lever system. Despite these differences, two arguments suggest that our experiments are informative: cutting forces of pristine mandibles were close to a theoretical minimum for PDMS; and although more complex mandible motion may decrease cutting forces in some cases [84,85], out-of-plane forces applied to thin sheets likely result in sheet bending instead of concentrating tensile stresses, and are thus more likely to increase rather than decrease cutting forces. Because leaf-cutter ants already need to show exceptional morphological and physiological adaptations to be able to produce bite forces sufficient to cut leaves [40,86], it is biologically implausible and physically impossible that forces during ‘free cutting’ are substantially amplified over the minimum force dictated by leaf toughness (see below for a detailed quantitative argument).

### (a) Size-invariance of cutting forces puts larger workers at an advantage

The weak size-dependence of cutting forces stands in stark contrast to the strong positive allometry of maximum bite forces in *A. vollenweideri*, which grow in almost direct proportion to body mass, *F*_b_ ∝ *m*^0.9^ [40]. As a result of this difference, the fraction of the maximum bite force required to cut the same material will decrease almost in direct proportion to mass, too, *F*_c_/*F*_b_ ∝ *m*^−0.9^—a factor of 30^0.9^ ≈ 20 across the size range considered in this study. For materials that could in principle be cut by workers across the size range, the differential scaling of bite and cutting forces affords considerable behavioural flexibility to larger workers, bound by two extreme scenarios.

First, larger workers may choose to bite with maximum force, i.e. fully activate their closer muscles during cutting. The excess force *F*_b_/*F*_c_ directly determines the maximum possible strain rate of the mandible closer muscle during cutting [87]; larger ants would then cut with substantially larger speeds. Cutting speed amplification may, however, be attenuated by viscoelastic effects that incur speed-dependent losses. In fracture, viscoelastic losses amplify the critical force by some power of the crack speed, and typically, $F_{f}\propto \sqrt{v_{c}}$ [88–90]. However, cutting forces usually show a much smaller speed-dependence, as the characteristic crack dimensions are tied to cutting-tool geometry instead (e.g. [68,75]). Indeed, a threefold increase in cutting speed resulted in an increase in cutting force of only 20%, compared with about $\sqrt{3}\approx 75\%$ expected for tearing ([68] and see electronic supplementary material, figure S1).

Second, and alternatively, large workers may choose to bite with the same multiple of the required cutting force as small workers, i.e. only sub-maximally activate the mandible closer muscle during cutting, in which case muscle strain rate would be approximately equal [87]. Although the cutting speed of larger ants would be sub-maximal as a result; this may be energetically advantageous, because muscle operates with maximum mechanical efficiency—the ratio between metabolic energy expended and mechanical energy produced—over a narrow range of intermediate strain rates [91]. On the basis of these arguments, we surmise that, even where a leaf can in principle be cut by small workers, it may be advantageous to assign larger workers to the task: they may cut either more quickly or with higher efficiency. In practice, foraging is a complex behaviour, and the behavioural choices of workers and their impact on the scaling of cutting speed and mechanical efficiency need to be addressed experimentally in future work.

### (b) Cutting force variation is mainly driven by mandibular wear rather than body size

Throughout their lifetime, leaf-cutter ant workers may cut a substantial amount of leaf tissue. As a rough estimate, a mature colony of about one million foragers cuts about 3000 m^2^ of leaf area per year [25], and each square metre requires *ca* 3 km of cutting [92]. Workers actively forage for about four months [50], and thus cut approximately 3 m leaf tissue, (4 × 3 × 3 × 10^6^)/(12 × 10^6^) = 3, or about 500 times their body length (*ca* 6 mm for a typical *A. vollenweideri* forager [93]). Such extensive leaf-cutting likely causes substantial mandibular wear [27]. Consistent with this conjecture is the observation that average cutting forces of pristine and forager mandibles differed by about 35 mN, or a factor of about 2 for PDMS sheets, comparable to results on leaf lamina reported for closely related *A. cephalotes* [27]. The absolute difference may sound small, but it amounts to about 50% of the force required to cut the median tropical leaf, and to about 15% of the maximum bite force of a medium 10 mg forager [8,40]. In light of the absence of a strong size-effect, it appears that most of the difference between pristine and forager mandibles stems directly from mandible wear. Indeed, even a simple empirical wear index, based on the weighted average length change of the two distal-most teeth, captures a remarkable 30% of the variation in cutting force, in striking contrast to the meagre 5–10% of variation explained by body mass (table 1).

The substantial effect of wear on cutting forces is biologically meaningful, for it implies that wear can compete with body size in determining the ability of a worker to cut a given substrate: cutting forces for mandibles from workers with a body mass between 4 and 6 mg varied by a factor of 7 (*n* = 14), equivalent to the difference in maximum bite force between two workers that differ in mass by about a factor of 7^1/0.9^ ≈ 8 [40]. The effect of wear can thus be as large as the effect of an eightfold reduction in the effective physiological cross-sectional area of the mandible closer muscle [52]. Both the susceptibility and the exposure to wear itself may be size-dependent, putting smaller workers at further disadvantage. Mandibles of smaller workers may be more susceptible to wear, because they have to exert similar forces, but are of smaller characteristic dimensions [12,27]; they may be more likely to be worn, because foraging parties tend to be dominated by ants of intermediate size (between 3 and 10 mg, Walthaus *et al.* 2023, in preparation). In support of these hypotheses, three lines of evidence may be presented. First, in large workers (body mass greater than 30 mg, *n* = 10), cutting forces varied only by a factor of 3 across pristine and forager mandibles, as opposed to a factor of 7 for the mandibles of small workers (body mass less than 6 mg, *n* = 24, see figure 2*a*). Second, although the scaling coefficients for total cutting forces of both callows and forager mandibles were not significantly different from zero (table 1), they were significantly different from each other (see Results). Third, both absolute and relative wear index tended to decrease with size, although these trends were not significant (see Results and electronic supplementary material, figure S3AB).

Based on the significant increase of cutting forces associated with mandible usage, we may speculate about the downstream effects of wear on the foraging performance of both small and large workers. Previous analysis of leaf mechanical properties, in combination with bite force experiments, suggested that a 30 mg worker may be able to cut almost all species of tropical leaves, whereas a 3 mg worker may only be able to cut about half of them [8,40]. Although this analysis neglected the effects of mandible geometry, it still serves as a reasonable starting point to estimate the effects of wear. We may calculate the reduction in the fraction of cuttable leaves resulting from wear based on the following two assumptions. First, the required cutting force for a pristine mandible, *W* = 0, is size-invariant and approximately equal to the product between fracture toughness and leaf lamina thickness (also see below). Second, the increase in cutting force with wear is material-independent, and equal to the regression slope extracted for PDMS (2.09 mN μm^−1^). For mandibles subjected to considerable wear, *W* = 20 μm, the minimum required cutting forces would thus be shifted up by *ca* 40 mN for all leaves. For a 30 mg worker, the fraction of cuttable leaves would be virtually unaffected (99.5%), whereas a 3 mg worker would now be able to cut fewer than 10% of tropical leaves, compared to almost 50% with pristine mandibles.

The significant increase of cutting forces with wear, and the conjectured reduction in cuttable plant leaves, likely necessitates behavioural adaptations, and may partially explain ‘age polyethism’, i.e. systematic changes in task preferences with worker age. Indeed, leaf-cutter ants with worn mandibles cut at significantly lower speeds, and are more likely to carry rather than cut [27]; the oldest colony workers may cease foraging altogether, and switch to mechanically less demanding tasks such as waste disposal [50,94]. The role of wear in determining the behavioural choices of leaf-cutter ants in particular and herbivorous insects in general is worthy of considerably more attention than it has received [44,53,55,57,60,95–97].

### (c) Biomechanics of cutting—how sharp are ant mandibles?

The size-invariance of cutting forces and their strong sensitivity to wear have biological consequences. From a mechanical perspective, both results may be surprising at first glance, and thus call for a more thorough evaluation. Intuitively, it appears reasonable to expect that mandibles of larger workers require a larger force to cut the same material. Indeed, the force required to fracture thin or thick model ‘targets’ with biological puncture tools increases significantly with characteristic dimensions of the tool, such as the tip diameter [12,14]. The expectation that tool size influences mechanical performance is closely tied to the notion of tool ‘sharpness’. However, a robust definition of sharpness as such is not a trivial task, as suitably illustrated by the large number of sharpness metrics suggested in the literature (e.g. [13,14,17,42,74,98–101]).

In order to rationalize our experimental results qualitatively and quantitatively, we first note that even an arbitrarily sharp mandible will not cut with arbitrarily small force. Cutting is akin to fracture, in the sense that it results in the creation of new surface area. Each unit area of new surface incurs an energy cost d*U*_*A*_, and the work that provides this energy has to be supplied by the externally applied load, so that, from a simple virtual work argument, d*U*_ext_ = d*U*_*A*_. Thus, and without loss of generality, the force *F* required to cut a slab of thickness *t* is bound from below by *F* ∼ *G*_c_*t*, where *G*_c_ is the energy per unit area of new surface, a characteristic material property [41,68,71,102]. For our experiments with PDMS, *G*_c_ ≈ 100 J m^−2^ and *t* ≈ 200 μm (see Methods), so that *F* ≈ 20 mN. This simple argument lends itself to a definition of an intuitive, quantitative and functionally relevant index for sharpness, *S*: the required cutting force is equal to the minimum possible force, and independent of tool geometry, if and if only the dimensionless group *S* = *G*_c_*t F*^−1^ is unity; the cutting tool may then be considered ideally sharp (for a conceptually similar suggestion, see [101]). The fracture forces measured for pristine mandibles of small workers are indeed very close to this theoretical minimum (figure 2*b*), suggesting that a further reduction in cutting force through changes in mandible morphology may not be possible. Thus, pristine mandibles of small workers appear ideally sharp, *S* ≈ 1, at least for PDMS (see below for a generalization of this argument). By contrast, pristine mandibles of larger workers, scalpel blades and the most worn mandibles of foragers have a functional sharpness index *S* between 2/3 and 1/5; in other words, cutting (and fracture) forces are between 50 and 500% larger than the theoretical minimum, hinting at contributions from cutting-tool geometry. The next task is thus to rationalize the putative influence of mandible geometry on cutting force.

The energy associated with the creation of new surface is not the only energy the external force has to supply. Friction, plasticity or sheet bending each carries its own energetic demands, so reducing the fraction of the external work available to drive the cut, d*U*_ext_ − d*U*_loss_ = d*U*_cut_ [102]. Some of these costs, for example due to elastic sheet bending or sidewall friction, can be accounted for by drawing the mandible through the cut again, and are thus removed in the fracture force (see figure 3*b* [11]); but others, related to the direct interaction between the mandible cutting edge and the material close to the crack tip, likely remain. The simplest possible assumption is that tool geometry can be characterized by a single characteristic length scale, *R* (e.g. [11,68,103–105]). From dimensional arguments, this length scale will then compete with a characteristic material length scale. In fracture mechanics, this material length scale is typically given by the ratio between *G*_c_ and a characteristic stress *σ*_c_, which may be interpreted physically as a critical crack tip opening displacement, or as the size of a crack process zone in which nonlinear mechanisms consume additional energy (e.g. [11,104,106–108]). Thus, for this simplest case, dimensional arguments suggest that the additional energy term will be of the form d*U*_loss_ ∼ *Cσ*_c_*Rt* d*x*, where *C* is a dimensionless constant. The fracture force now reads:

4.1 $$F_{f}=G_{c}t+C\sigma_{c}Rt,$$

from which the functional sharpness index follows as:

4.2 $$\frac{1}{S}=1+C\frac{\sigma_{c}R}{G_{c}}.$$

In both equations, the first term represents the unavoidable cost arising from fracture alone; the second term accounts for additional costs linked to tool geometry. For simple geometries such as a cylindrical wire, an exact analysis is possible, and yields *C* = (1 + *μ*), where *μ* is the coefficient of friction [41]. For our experiments, we equate *σ*_c_ with the ultimate tensile strength of PDMS (about 4 MPa [70]), and assume that the friction coefficient of mandibles on PDMS is similar to values for steel on PDMS, *μ* ≈ 1 [109,110]. The geometry-dependent term $2\sigma_{c}RG_{c}^{-1}$ then accounts for half of the cutting force, *S* = 0.5, if the characteristic length is $R=1/2G_{c}\sigma_{c}^{-1}=12.5\;\mu m$. A typical choice for *R* is the radius of the cutting edge (e.g. [11,12,14,68,98]), and indeed, our rather approximate calculation is in remarkable agreement with direct measurements of the cutting edge radius of worn mandibles in *A. cephalotes*, *R* ≈ 17 μm [27]. Pristine mandibles, in turn, may have a cutting edge radius as small as 50 nm [27], so that *S* = 0.996 ≈ 1, in seeming agreement with the observation that the pristine mandibles of the smallest workers approach the theoretical minimum cutting force for PDMS (figure 2*b*). The simple definition of sharpness suggested in equation (4.2) thus has the advantages that it is based on mechanical analysis instead of empirical correlation with observed mechanical performance, that it clearly separates material- and tool-dependent contributions to sharpness, and that its magnitude has a clear physical interpretation.

From this cursory analysis, we may surmise that fracture forces are effectively independent of mandible geometry if $2\sigma_{c}RG_{c}^{-1}\ll 1$, but grow in proportion to *R* ∝ *m*^1/3^ for $2\sigma_{c}RG_{c}^{-1}\gg 1$ [68,103]. These limits thus delineate two regimes characterized by geometric invariance and length scaling of cutting forces, respectively, and in practice, the scaling of cutting forces with *R* may fall anywhere in between. This result may be put to use in two ways.

First, and in combination with our experimental data, it allows an approximate assessment of the parsimonious but unverified hypothesis that the characteristic mandible dimension *R* is isometric, i.e. *R* ∝ *m*^1/3^. Plausible alternative hypotheses may be derived. For example, the tip radii of some insect claws depart from isometry and scale as *R* ∝ *m*^1/2^, presumably to ensure that tip stresses remain size-invariant [111]. In direct analogy, it is conceivable that pristine mandible cutting edge radii show a scaling shallower than isometry, or are even size-invariant. To test the hypothesis of isometry, we estimate the cutting edge radius *R* from the cutting force measured for a pristine mandible of the largest workers (40 mg in body mass), via equations (4.1) and (4.2), yielding *R*_40_ ≈ 5 μm. Next, we use this result to extract a proxy for the proportionality constant *a*, invoking the null hypothesis of isometry, *R* = *am*^1/3^, and then predict the variation of cutting force across the callow size range from 2.1 to 46.4 mg, using equation (4.1). An OLS regression on log_10_-transformed predictions yields an intercept of 1.33 and a slope of 0.07, remarkably close to the experimental values of 1.32 and 0.09 (units: mN, mg; see table 1). Our experimental results are thus consistent with isometry of the mandible cutting edge radius. Although *R* may vary by as much as a factor of 30^1/3^ ≈ 3 across the size range investigated in this study, cutting forces vary only little with size, because even large mandibles satisfy $2\sigma_{c}RG_{c}^{-1}<1$. However, the considerable variation in our data even for pristine mandibles limits the statistical power to establishing consistency, and direct experimental assessment, for example via scanning electron microscopy [12,27], is necessary to firmly establish isometry.

Second, equation (4.1) can be put to work to assess whether the size-invariance of cutting forces observed for a synthetic material such as PDMS may extend to natural materials typically cut by leaf-cutter ants. To this end, we extract proxies for the median *G*_c_ ≈ 400 N m^−1^, *t* = 200 μm and *σ*_c_ ≈ 3 N mm^−2^ from an extensive study on the leaf lamina of about 1000 tropical plant species [8], and again use equation (4.1) to predict the expected scaling of cutting forces. We find an intercept of 1.9 and a slope of 0.02. Thus, the size-dependence of the net cutting force in natural materials may be even weaker than for PDMS, because leaves have a higher toughness, but similar ultimate strength, so that $2\sigma_{c}RG_{c}^{-1}<1$, and the geometry-independent term in equation (4.1) dominates. We stress that this analysis is approximate, and cutting of plant leaves may for example incur larger bending costs, because they are much stiffer. Preliminary support is, however, available from cutting force measurements with laurel leaves. Based on the median tropical leaf with *G*_c_ = 400 N m^−1^, and *σ*_c_ = 3 N mm^−2^, and the lamina thickness of laurel, *t* ≈ 250 μm, equation (4.1) predicts cutting forces for a pristine mandible with *R* = 5 μm and a worn mandible with *R* = 12.5 μm of 108 and 119 mN, respectively, in reasonable agreement with our experimental results (figure 3*a*). Thus, a difference in cutting edge radius that would increase cutting forces in PDMS by about 40% increases those for the median leaf by a mere 10%. Although the simple model based on dimensional arguments appears to quantitatively capture salient features of our experimental data, more thorough experimental validation, including cutting measurements with a range of natural materials and direct measurements of cutting edge radii, are in order.

The putatively weak size-dependence of mandible cutting forces for natural materials has two consequences worthy of brief discussion. First, it implies that mandible wear needs to be more severe in order to have an appreciable effect on cutting forces. As an illustrative example, *S* = 0.5, corresponding to a doubling of the required cutting force, occurs for *R* ≈ 12.5 μm in PDMS; the equivalent radius for the median tropical leaf is *R* ≈ 67 μm—about five times larger. However, there is robust evidence that wear affects leaf-cutter ant performance even when cutting natural materials: the average fracture force required to cut laurel leaf lamina with forager mandibles was about 30 mN higher than for callow mandibles (figure 3*a*), and similar results were reported by Schofield *et al.* [27] for *A. cephalotes* workers and *Prunus lusitanica* leaves; leaf-cutter ants with worn mandibles cut at significantly lower speeds ([27], see also [53] for similar results on leaf beetles); and leaf-cutter ants with worn mandibles have different task preferences [27,50,94]. Clearly, the role of wear in modulating cutting forces of natural materials requires further experimental investigation. Second, and conversely, it suggests that even moderately small cutting edge radii may suffice to achieve *S* ≈ 1. For example, for *R* = 1 μm, *S* = 0.99 ≈ 1, and even for *R* = 10 μm, *S* = 0.87, still within 15% of the maximum sharpness for cutting the median leaf. Thus, selection pressure on material properties and edge geometry for the cutting tools of small animals may be less strong than previously suggested [12,47].

## 5. Conclusion and outlook

The ability to cut leaves involves complex interactions between worker size, bite force capacity, wear-dependent cutting forces, plant-material properties and adaptive cutting behaviour. We tried to untangle this complexity, by removing the confounding effects of material heterogeneity and nonlinear mandible motion, and studied the effects of mandible size across two experimental groups with varying levels of mandibular wear.

Although smaller ants may experience a larger increase in cutting force from pristine to worn mandibles, cutting forces were nevertheless approximately size-independent, in contrast to our initial hypothesis. The ability to cut leaves is thus mostly affected by size-dependent bite forces, plant-material properties and mandibular wear. As a result, larger ants only need to apply a substantially smaller fraction of their maximum bite force to cut the same material. In agreement with our second hypothesis, the effects of wear on cutting force can be substantial, which may strongly reduce the range of accessible plant tissues for small workers.

Pristine mandibles of callow workers are exceedingly ‘sharp’, and even mandibles with moderate levels of mandibular wear cut with similar forces to the ‘sharpest’ pristine scalpel blade; these results indicate morphological adaptations of leaf-cutter ant mandibles to the high mechanical demands of cutting [27,45].

A natural extension to this work will be to use other materials as cutting substrate, and to test quantitative predictions on cutting force variation and cutting edge geometry for a broader selection of biologically relevant substrates. A careful inspection of the mandibular cutting blade, in combination with mandible abrasion experiments, may reveal structural and chemical adaptations to increase mandibular wear resistance [43,45].

We hope that the findings of this study will help to increase our understanding of size-specific foraging preferences in leaf-cutter ants, and more generally, may provide a framework to investigate the relative importance of tool geometry versus material properties in biological cutting.

## Data Availability

The data are provided in the electronic supplementary material [112].
